# Editorial Extracts and Gleanings

**Published:** 1874-02

**Authors:** A. R. Kilpatrick

**Affiliations:** Texas


### Editorial Extracts and Gleanings.

BY A. R. KILPATRICK, M.D., OF TEXAS.

*Malvaviscus Drummondii, Spanish Apple.*—This is a native plant in the Brazos swamp of Texas, and is extensively used, both by physicians and the laity, to form demulcent, mucilaginous drinks in fever. It is also used in the form of poultice, by pounding the root into a pulpy mass, and furnishes an agreeable dressing where a soothing application is needed. It belongs to the Mallow or *Malvaca* family, and possesses properties common to them; beneficial in nephritic, cystic or urethral complaints.

It grows mostly in bottom land, has a slender stem, more like a vine; pale green, hairy leaves, with beautiful red flowers. It is frequently transplanted to yards and gardens, both for the beauty of the flowers and the medical properties of the roots, which are large and fleshy, yielding great quantities of mucilage, simply by slicing and placing in a pitcher of cold water.

*Modiola Multifida.*—Move weed—is another plant common in Texas, and extensively used for poultices, on account of its demulcent mucilaginous qualities. It looks very much like the ground ivy, continues green in the winter, and is used in place of slippery elm, which is not plentiful here.

*Liquidambar Styraciflua*—Sweet-gum—which is so plentiful in the Southern States, is only found occasionally in Texas. Liviodendron Tulipiera—Poplar—is never seen.

*Bites of Snakes and Stings of Scorpions, Centipedes, Insects, Spiders, etc.*—As our journal is read all over the South, where persons are frequently poisoned by reptiles and vermin, we know they will be glad to find some reliable remedy of easy application in such emergencies. The common spirits of turpentine and the tincture of lobelia inflata, used locally and internally in quantities and doses suited to the age and sex, and the violence of the case, will give relief.

To our readers in Texas, it no doubt will be good news to know that Dr. Gideon Lincecum, of Long Point, Washington county, Texas, announces that the common trailing plant, called Devil's shoe-string, or Turkey-pea, or Goat's rue. *Tephrosia Virginiana* is a certain and safe remedy for the sting of the Centipede, which is considered one of the most poisonous vermin in the country. The technical name by which it is known to naturalists, is, *Myriapoda Scolopendra,* and by the ignorant, is often called Santafee. The Goat's rue is used in strong decoction, both internally and externally directly to the diseased part. It can be also used by bruising the weed, squeezing out the juice and giving it in sweet milk.

*Small-pox in Houston, Texas.*—This disease, after being in the city about three weeks, has been traced to a man from Longview, a railroad town in Upshur county, where the small-pox prevailed last spring and summer, and where he had had the disease. He came down the Great Northern Railroad and landed at Union Depot, where he left his clothes, and some persons with whom they were entrusted, took the small-pox from them. He was shaved at a barber shop near the Hutchin's House, and had a bath, and the boy who waited on him and brushed his clothes, also took the disease. From these different points the disease spread, mostly amongst the colored population, although several whites have had it, and in all, there have been sixty cases and sixteen deaths up to the 21st Jannary, 1874.

The disease is of a violent type, most of the cases being confluent. The Mayor of Houston has been remiss in his duties in allowing the cases to remain in the city. Pest houses have been provided beyond the city limits, and vaccination is going on as fast as possible.

*Prophylactic Treatment of Intermittent Fever.*—In the *Philadelphia Medical and Surgical Reporter* for January 3, 1874, Dr. James Y. Sheaver gives a brief article recommending the constant use, three times a day of tincture Iodine in doses ranging from one to ten drops, in proportion to the age and sex of the patient, for the prevention of a return of the chills, after they have been checked with sulphh. quinine. Give in a tablespoonful of cold water, before meals. He has had remarkable and encouraging success in following this course with cases in a highly malarious region, at Sinking Springs, Burks county, Pa., in which all other antiperiodics had failed to keep off a return of the chills.

*Cerebro-spinal Meningitis.*—Dr. James Gillespie, of Abbeville, Ala., gives in the *Medical and Surgical Reporter*, of Philadelphia, for December 27th, a brief statement of his treatment of this formidable disease. He reports only one case which he treated, although he intimates that he has used the remedy with uniform success for sometime. He gives by enema, from fifteen to twenty drops of the *tincture of veratrum viridi in two ounces* of water. If that does not relax the muscles, he repeats the enema in half an hour, or until there is a thorough relaxation of the whole system. He says the veratrum seems to act specifically. It relaxes the muscles, disgorges the capillaries and equalizes the circulation. Should the medicine cause nausea and vomiting, a little brandy or whiskey soon removes it, and these are also given by enema if the patient is unable to swallow. As soon as the patient's stomach can tolerate medicine, he gives large doses of quinine, from fifteen to twenty grains every four hours, also by enema if necessary, to prevent a return of the paroxysm. As an adjeuvant he *pours* cold water on the head. He eschews sinapisms, cups, blisters, liniments, etc., as all humbug.

*Medical Signs and Doses.*—It is proposed in many publications of the day, that some change should be made in the signs used in the dispensing and prescribing medicines. This should be freely discussed now, so that at the next meeting of the American Medical Association, some definite action may be taken in the premises.

Many disastrous mistakes have occurred from the great resemblance in the written characters for *drachms* and *ounces*, and occasionally from the bad writing of the marks *gr.* and *gtt.*

There should be a total dissimilarity between the drachm and ounce signs. Let those who have a taste for such things, set their ingenuity at work on the problem.

Another question is now being agitated in British Medical Journals and Local Societies, viz: the invention and adoption of a sign to be used by medical men to mark unusual doses in prescriptions. An unusual dose is one in the excess of the maximum adult dose of the Pharmacopœia, or a dose exceeding that usually administered. Valuable time is sometimes lost, resulting in detriment to the patient, arising from hesitation and doubt on the part of the dispenser of the medicine, which could all readily be avoided by the needed sign, to assure him that the dose has not been *mis*-prescribed.

*A New Method of Producing Local Anæsthesia..*—Dr. Horvath, of Keiff, has lately proposed (The Doctor) a new method of producing local anæsthesia. If the hand be immersed for a short time in ice-water, severe pain is caused. But in experiments made in reducing the temperuture of frogs by means of cold alcohol, Dr.

Horvath found that no such pain was produced when the hand was immersed in cold alcohol, not even when the temperature of the alcohol was as 5° C. Glycerine was found to possess a similar property. Ether caused pain, and quicksilver more acute pain still, causing the speedy withdrawal of the finger when plunged into this liquid at a temperature of 3°. It was next ascertained that when the finger was held for quite a long time in alcohol having a temperature of 5° C. no pain was experienced. Moreover, although the faintest touch was distinctly perceived in his finger, no pain was experienced from sharp pricks. This seemed to show that the application of cold alcohol has the effect of depriving the part of the special sensibility to pain, without, however, impairing the delicacy of the tactile sensation, which, as is well known, resides in the superficial integument. This apparent possibility of the artificial separation of these two nervous functions—vis., the tactile sensation and the sensation of pain, and the temporary suspension of the latter—seemed important in a physiological point of view, and also of no small practical utility in allaying certain forms of local pain, more especially that caused by burns and surgical operations. Dr. Horvath had an opportunity of testing the value of this application to burns on his own person as well as upon others; and not only was all pain instantly allayed directly the part was immersed in acohol, but it was found that the wound very speedily began to assume a more healthy appearance, the surrounding redness rapidly failing.—*Practitioner.*

*Vehicle for the Administration of Chloroform.*—A French medical journal remarks that the best course is to dissolve the chloroform in glycerine (1; 2), which is effected with tolerable facility, and gives a very clear solution, pleasant to the taste, and with strong odor of chloroform. This solution can be mixed in all proportions with water without the occurrence of any precipitation, though the odor is distinctly perceptible. In forming the mixture, it is well to add the chloroform slowly, and to mingle the two thoroughly. It should be left at rest for twenty-four hours; at the expiration of this period, a portion of the chloroform will be found to have collected at the bottom of the vase; this should be separated and mixed with additional part of glycerine, when no farther separation will occur. This mixture may be kept for some time without any loss of chloroform by evaporation.—*Boston Journal of Chemistry.*

